# Intraductal Radiofrequency Ablation Followed by the Stent-in-Stent Technique for the Removal of an Embedded, Intraductally Migrated Fully Covered Biliary Metal Stent: A Case Report

**DOI:** 10.7759/cureus.119303

**Published:** 2026-11-08

**Authors:** Felipe S Santos, André O Ardengh, Vitor H Lopes, Renato Baracat, Fauze Maluf-Filho

**Affiliations:** 1 Gastroenterology and Endoscopy, Hospital das Clínicas da Faculdade de Medicina da Universidade de São Paulo (HC-FMUSP), São Paulo, BRA; 2 Gastroenterology, Cancer Institute of the State of São Paulo, Hospital das Clínicas da Faculdade de Medicina da Universidade de São Paulo (HC-FMUSP), São Paulo, BRA

**Keywords:** benign biliary stricture, biliary stent migration, cholangioscopy, embedded biliary stent, endobiliary radiofrequency ablation, ercp, fully covered metal stent, stent-in-stent technique

## Abstract

Fully covered self-expandable metal stents (FCSEMSs) are widely used in benign biliary strictures because they combine sustained radial force with the possibility of later endoscopic removal. When the stent stays in place for too long, tissue hyperplasia and intraductal migration can leave the stent firmly embedded in the duct wall. We report a 38-year-old woman with a Strasberg E2 bile duct injury after cholecystectomy and a benign segmental stricture of the common hepatic duct, treated with dilation, plastic biliary stents followed by a 10 mm × 6 cm FCSEMS. She was subsequently lost to follow-up for two and a half years and returned with mild abdominal pain and cholestasis. By then, the stent had migrated intraductally, with its distal end approximately 2 cm above the major papilla, and was impacted by granulation tissue. Conventional retrieval failed, as did a stent-in-stent attempt with a 10 mm × 8 cm FCSEMS. Cholangioscopy showed granulation tissue within the lumen and at the extremities of the impacted stent. Intraductal bipolar radiofrequency ablation (RFA) was applied at 9 W for 60 seconds along the entire length of the stent using a Habib EndoHPB catheter (Boston Scientific, Marlborough, MA, USA). Hyperplastic tissue was visibly reduced, but the stent could still not be extracted in the same session, so the stent-in-stent technique was repeated with a 10 mm × 8 cm FCSEMS and an 8.5 Fr × 12 cm plastic biliary stent. Finally, 49 days later, the embedded stent had mobilized toward the major papilla and was retrieved with foreign-body forceps. A small, low-output fistula of the mid common bile duct was identified on occlusion cholangiography. It was successfully managed with a further FCSEMS and a plastic stent. Sequential intraductal RFA followed by delayed stent-in-stent placement may serve as a rescue strategy when an embedded biliary FCSEMS is not removable by conventional means or by the stent-in-stent technique alone.

## Introduction

Fully covered self-expandable metal stents (FCSEMSs) are an established option for selected benign biliary strictures. Their large diameter provides sustained radial force, and the covering is intended to prevent tissue ingrowth and keep the stent removable [1,2]. European Society of Gastrointestinal Endoscopy guidelines recommend temporary placement of either multiple plastic stents or an FCSEMS in this setting [1].

Removability, however, is not always guaranteed. Migration is one of the most relevant adverse events associated with these devices, and prolonged indwelling may lead to hyperplastic tissue growth through and around the mesh, embedding the stent in the bile duct wall [2,3]. Proximal migration and hyperplastic tissue overgrowth are the two mechanisms most often reported behind failed FCSEMS removal in benign biliary disease [4].

The stent-in-stent technique is the usual rescue maneuver in this situation: a second FCSEMS is deployed inside the embedded stent to compress the hyperplastic tissue and allow removal at a later session [4-7]. Endobiliary radiofrequency ablation (RFA) has recently been described as an adjunct in this scenario, including in patients in whom a stent-in-stent attempt had already failed [8].

We report a patient with an intraductally migrated and embedded FCSEMS after prolonged indwelling for a benign postoperative biliary stricture, in whom conventional retrieval and a first stent-in-stent attempt had both failed, and in whom endoscopic removal was ultimately achieved with intraductal bipolar RFA followed by repeat stent-in-stent placement.

## Case presentation

A 38-year-old woman had a Strasberg E2 bile duct injury [9] sustained during laparoscopic cholecystectomy. She presented with cholestasis two months after surgery and was diagnosed with a benign segmental stricture of the common hepatic duct. She subsequently underwent endoscopic treatment with stricture dilation and placement of plastic biliary stents, without resolution of the stricture. A 10 mm × 6 cm FCSEMS was then placed, with the intention of removing it after six months.

She did not attend the scheduled endoscopic reassessment and was lost to follow-up for two and a half years. She presented again with mild abdominal pain and cholestasis, with elevated serum alkaline phosphatase and gamma-glutamyl transferase levels (Table 1). The FCSEMS had migrated intraductally, with its distal end lying approximately 2 cm above the major papilla, and had become impacted by granulation tissue growing through the mesh. Conventional retrieval was unsuccessful. A stent-in-stent attempt was then made by deploying a 10 mm × 8 cm FCSEMS inside the impacted stent. Six weeks later, at the following endoscopic retrograde cholangiopancreatography (ERCP), the internal 10 mm × 8 cm FCSEMS and the previously placed plastic biliary stent were easily removed, while the original 10 mm × 6 cm FCSEMS remained embedded. Cholangioscopy was performed to establish the reason for impaction and demonstrated granulation tissue within the lumen and at the extremities of the stent (Figure 1).

**Table 1:** Laboratory findings at re-presentation Reference ranges refer to adult women.

Parameter	Patient value	Reference range (adult female)
Alkaline phosphatase (ALP)	378 U/L	35-104 U/L
Gamma-glutamyl transferase (GGT)	416 U/L	5-36 U/L

**Figure 1:**
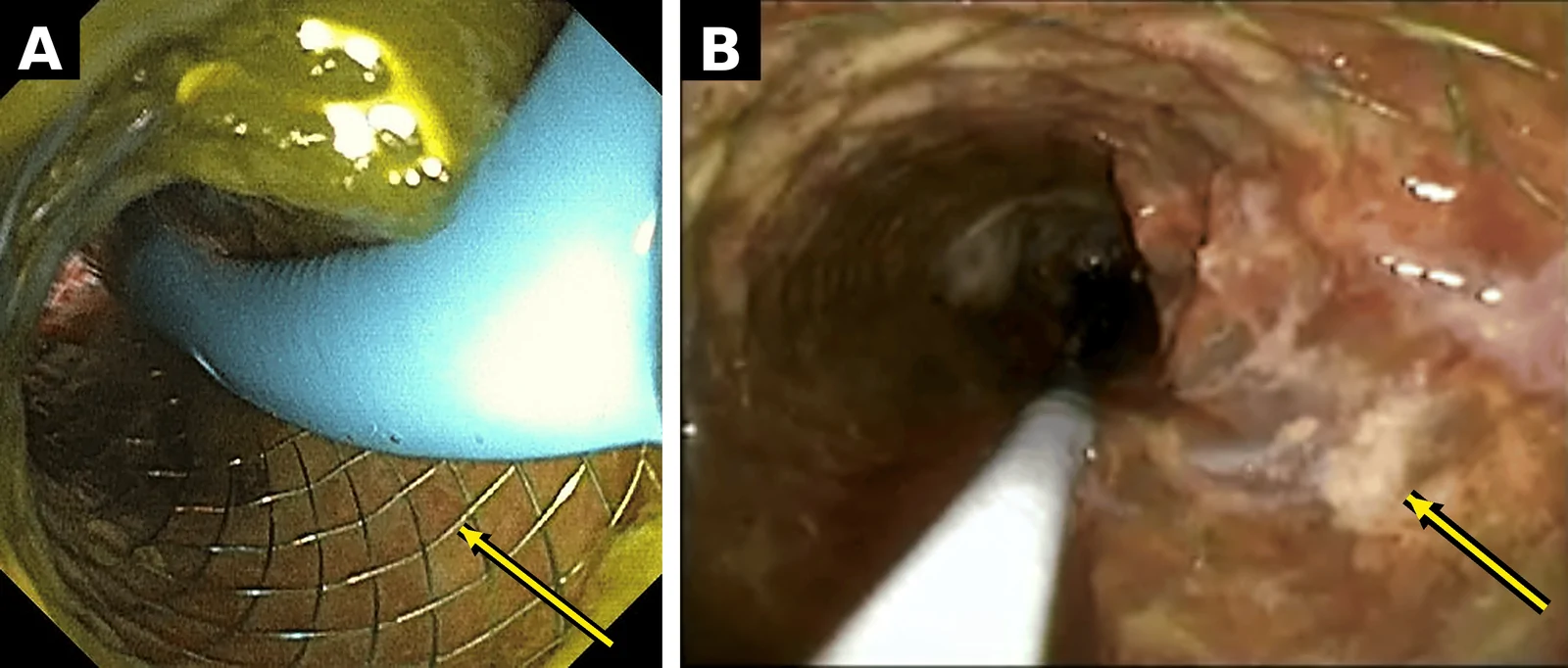
Endoscopic and cholangioscopic appearance of the embedded fully covered self-expandable metal stent (A) Endoscopic view obtained with the duodenoscope, showing the cholangioscope emerging from the working channel and entering the bile duct; the arrow indicates the impacted fully covered self-expandable metal stent. (B) Cholangioscopic view of the bile duct lumen; the arrow indicates granulation tissue at the end of the impacted stent.

Because mechanical compression alone had already failed, intraductal bipolar RFA was performed using a Habib EndoHPB bipolar radiofrequency catheter (Boston Scientific, Marlborough, MA, USA) at 9 Watts for 60 seconds along the entire length of the impacted stent (Figure 2). Hyperplastic tissue was visibly reduced after ablation, but the stent remained embedded.

**Figure 2:**
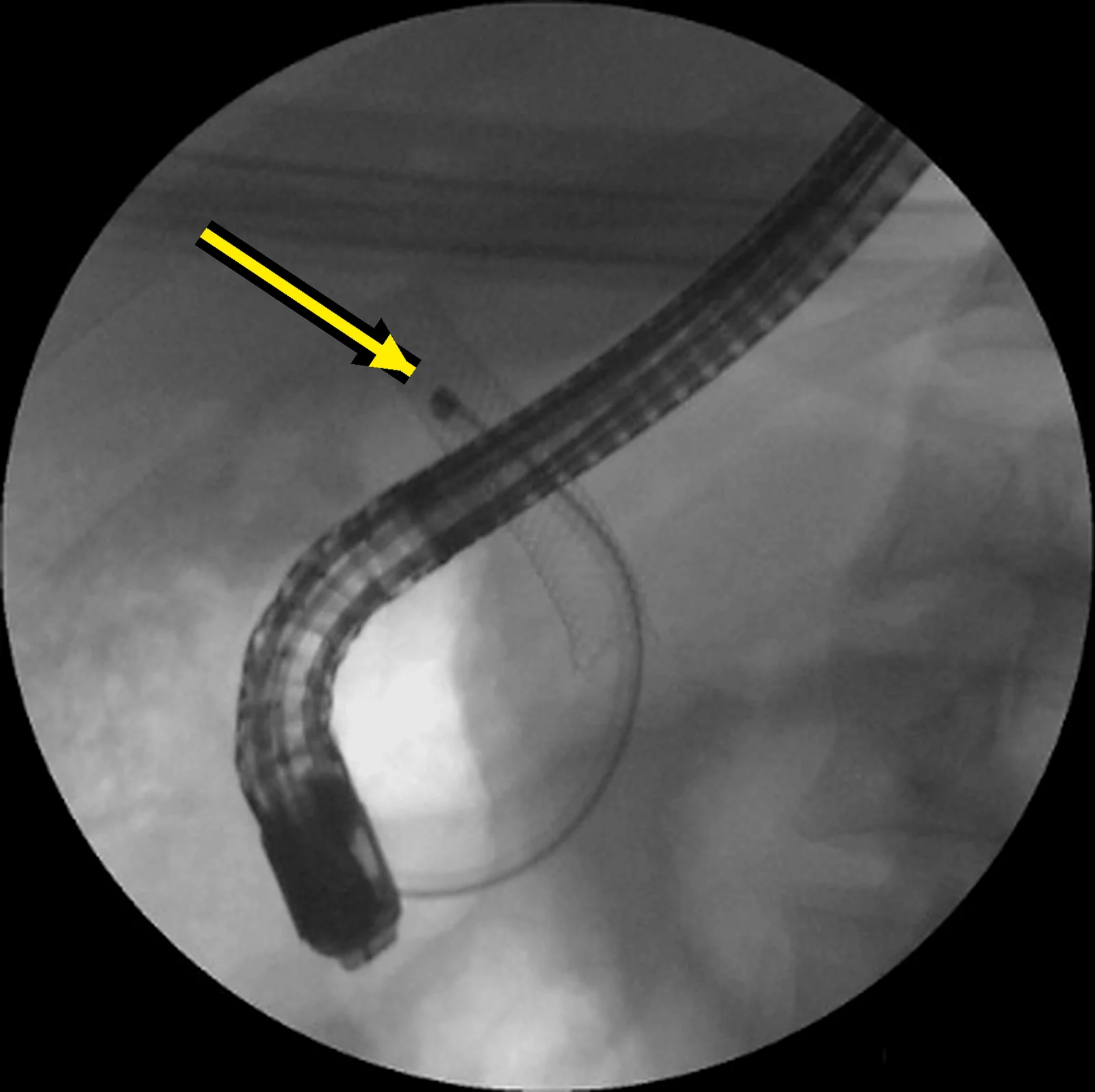
Intraductal radiofrequency ablation of granulation tissue associated with the embedded biliary stent Fluoroscopic image obtained during intraductal bipolar radiofrequency ablation, performed at 9 W along the entire length of the impacted stent. The arrow indicates the tip of the radiofrequency catheter within the impacted stent.

The stent-in-stent strategy was therefore repeated, with deployment of a 10 mm × 8 cm FCSEMS inside the impacted stent together with an 8.5 Fr × 12 cm plastic biliary stent (Figure 3).

**Figure 3:**
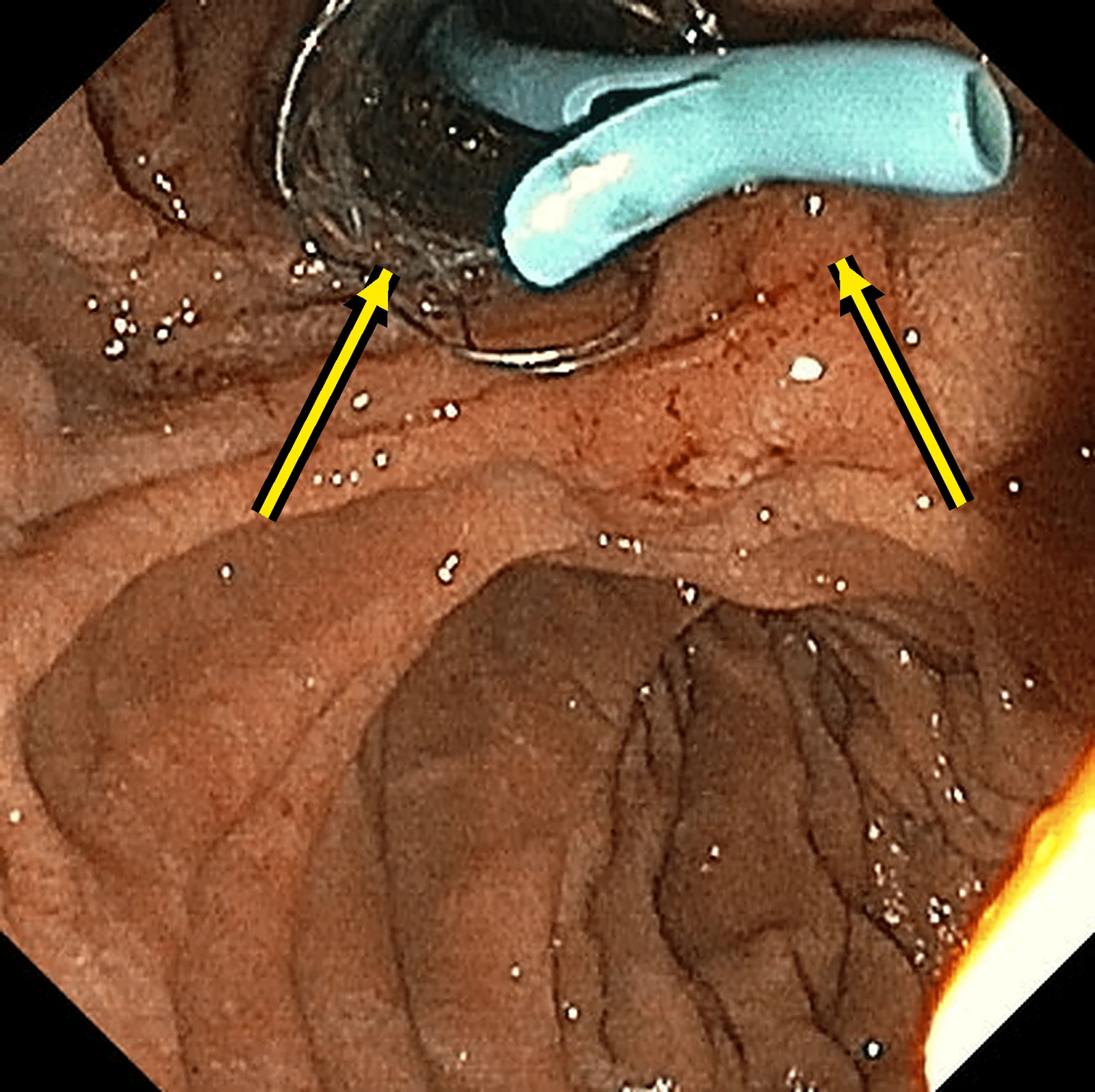
Repeat stent-in-stent placement after radiofrequency ablation Endoscopic view of the major papilla after deployment of a 10 mm × 8 cm fully covered self-expandable metal stent inside the impacted stent (left arrow), together with an 8.5 Fr × 12 cm plastic biliary stent (right arrow).

Repeat ERCP was performed 49 days later. The internal stents were removed, and balloon sweeping of the common hepatic and common bile ducts retrieved biliary sludge, a stone, and amorphous material. The previously impacted FCSEMS had mobilized toward the major papilla. It was grasped with foreign-body forceps and finally removed, although some resistance was encountered during extraction (Figure 4).

**Figure 4:**
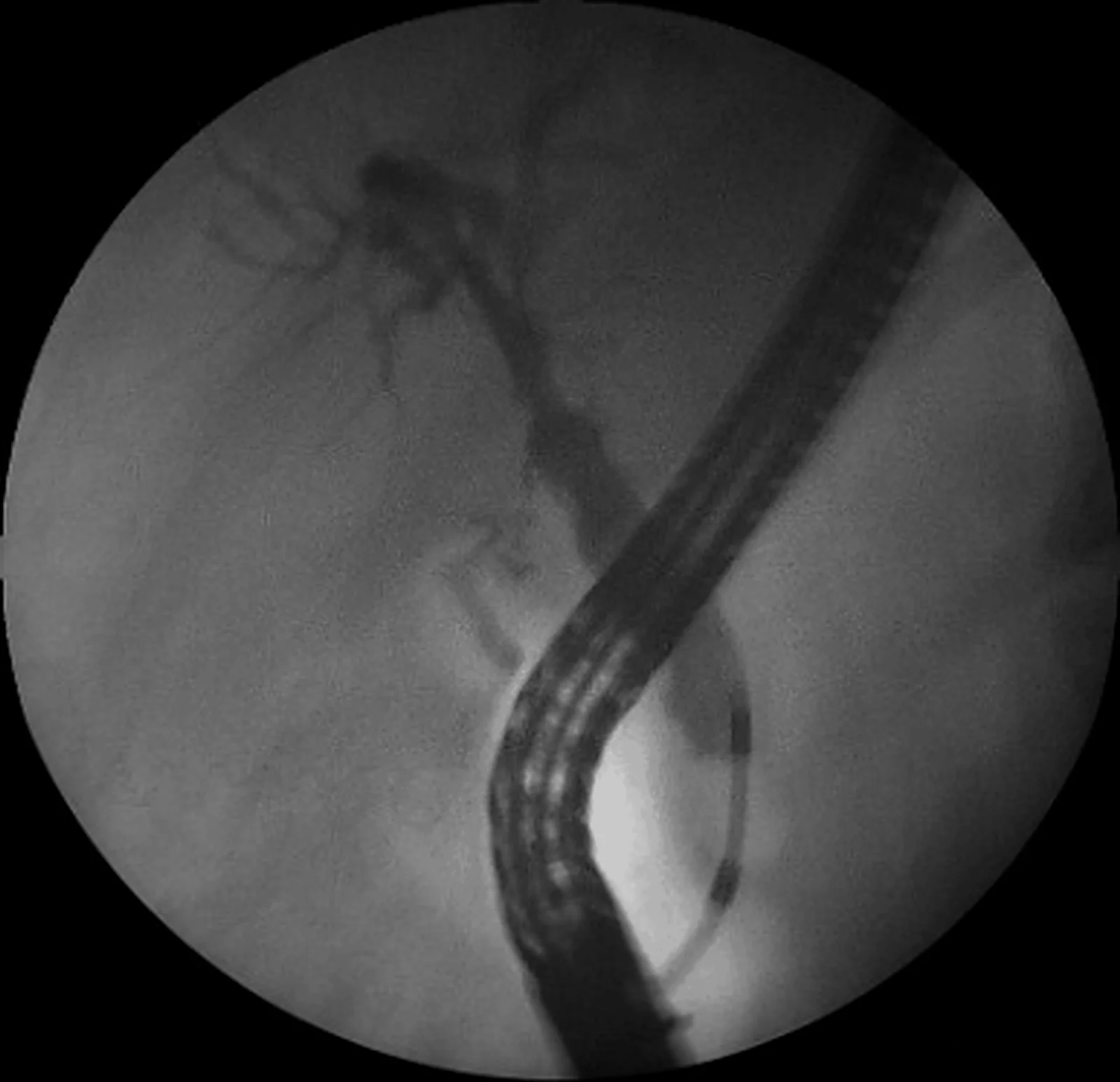
Repeat endoscopic retrograde cholangiopancreatography 49 days after repeat stent-in-stent placement Fluoroscopic image obtained during the repeat procedure, at which the previously impacted stent had mobilized toward the major papilla and was retrieved with foreign-body forceps.

An occlusion cholangiogram obtained after stent removal demonstrated a small, low-output biliary fistula in the mid common bile duct. A 10 mm × 8 cm FCSEMS was placed across the fistula together with a 10 Fr × 12 cm plastic biliary stent. The patient had no procedure-related adverse events. She remains in outpatient follow-up, with removal of the newly placed metal stent planned; at the time of writing, this procedure had not yet been performed.

## Discussion

FCSEMSs are attractive in benign biliary strictures because a single device delivers the dilating force of several plastic stents and, in principle, can be removed once the stricture has been remodeled [1,2]. This approach is supported by prospective data: a large multinational study evaluated FCSEMS removal after extended indwelling and the durability of stricture resolution [10], a randomized trial showed that a single FCSEMS was non-inferior to multiple plastic stents for stricture resolution while requiring fewer ERCPs [11], and long-term outcomes have also been reported specifically for post-cholecystectomy strictures [12]. In these studies, however, stents were removed according to a planned schedule. In our case, the consequences of a 30-month indwelling time were precisely those described in the literature: intraductal migration and hyperplastic tissue growing through the mesh until the stent was fixed to the duct wall [2,3]. Familiari et al. showed early on that impaction, tissue ingrowth, and migration are the main reasons a biliary self-expandable metal stent (SEMS) becomes difficult to retrieve [3]. Removal of FCSEMSs is generally safe when performed as planned [13], but covering reduces rather than abolishes the risk of embedding when the stent-tissue interface has had years to mature.

Cholangioscopy identified granulation tissue as the cause of impaction, supporting a strategy directed at the tissue rather than further mechanical traction.

When conventional extraction fails, the stent-in-stent technique is the established next step. A second FCSEMS deployed inside the embedded stent compresses the hyperplastic tissue and may induce pressure necrosis, so that the original stent loosens before a further attempt [4-7]. Menon reported this for an embedded covered biliary SEMS [5], Arias Dachary et al. and Tan et al. described the same principle applied to uncovered devices [6,14], and Tringali et al. reported five patients with benign biliary strictures in whom FCSEMS removal had failed because of proximal migration or tissue overgrowth, and in whom placement of a second FCSEMS allowed successful extraction in every case [4]. More recent data are less uniformly favorable. Komori et al. reviewed 17 patients with embedded biliary metal stents and reported an overall technical success rate of 76.5%; removal succeeded in all cases in which embedding was related to hyperplasia, and most failures occurred when extraction was attempted within four weeks of placing the second stent [7].

In our patient, a stent-in-stent attempt had already failed. Endobiliary RFA has been used to restore the patency of biliary metal stents occluded by tissue ingrowth [15]. Intraductal bipolar RFA was therefore applied along the full length of the embedded stent to treat the granulation tissue, using a bipolar catheter at 9 W, which lies within the 7-10 W range generally reported for this device [16]. Ablation clearly reduced the tissue burden but did not make immediate extraction possible. Experimental data offer a plausible explanation: the depth of bipolar ablation is markedly reduced inside a metal stent, particularly when the electrodes are in contact with the stent wires [17,18]. RFA should not be expected to free a chronically embedded stent in a single session. Placing a further FCSEMS inside the ablated stent and waiting seven weeks allowed both mechanisms to act, and by then the stent had loosened and mobilized distally enough to be grasped with foreign-body forceps. This interval is also consistent with the observation of Komori et al. that delayed rather than early reintervention improves the chance of success [7].

The sequence used here mirrors the approach recently reported by Lambin et al., who applied endobiliary RFA to tissue ingrowth in seven patients with embedded biliary metal stents and then systematically placed an internal FCSEMS; three of these patients had already failed a stent-in-stent attempt, and stent removal was ultimately successful in five of seven [8]. Their series consisted of men with predominantly malignant disease, whereas our patient was a young woman with a benign postoperative stricture, an exceptionally long indwelling time, intraductal migration, and granulation tissue documented on cholangioscopy. The same combination proved effective in a clearly different clinical setting, which we believe adds to the limited experience available.

The small, low-output fistula found on occlusion cholangiography after removal is a reminder that extracting a stent embedded for years is not a trivial maneuver. The duct wall in contact with the mesh is inflamed and thinned, and the resistance encountered during extraction carries a real risk of perforation or avulsion. In this case, the leak was small and was managed endoscopically with an FCSEMS across the affected segment together with a plastic stent, without clinical consequences.

This report has limitations. Because RFA was followed by a second FCSEMS and a seven-week delay before extraction, the contribution of ablation cannot be separated from that of prolonged mechanical compression, and progressive loosening over time cannot be excluded. The optimal power setting, duration, and number of RFA applications for this indication also remain undefined. Follow-up is still short: the patient has not yet returned for removal of the currently indwelling stent, so neither resolution of the stricture nor the removability of that stent can yet be reported. Prospective data are needed before this combination can be recommended as anything other than a rescue option.

## Conclusions

FCSEMSs are designed to be removable, but prolonged indwelling, intraductal migration, and tissue hyperplasia can embed them firmly enough to defeat conventional retrieval and even a first stent-in-stent attempt. In this patient, cholangioscopy identified granulation tissue as the cause of impaction; intraductal bipolar RFA ablated that tissue, and repeat stent-in-stent placement with delayed extraction seven weeks later allowed the stent to be removed endoscopically, avoiding surgery. Sequential RFA and stent-in-stent placement appears to be a reasonable rescue option in selected patients with difficult biliary metal stent removal, although its safety, efficacy, and optimal technical parameters remain to be defined. The case is also a reminder that FCSEMS can embed in the biliary tree depending on the indwelling time. 
